# The Importance of Stem Photosynthesis for Two Desert Shrubs Across Different Groundwater Depths

**DOI:** 10.3389/fpls.2022.804786

**Published:** 2022-03-10

**Authors:** Ran Liu, Xiaolong Feng, Congjuan Li, Jie Ma, Yugang Wang, Yan Li

**Affiliations:** ^1^State Key Lab of Desert and Oasis Ecology, Xinjiang Institute of Ecology and Geography, Chinese Academy of Sciences, Urumqi, China; ^2^Fukang National Station of Observation and Research for Desert Ecosystem, Xinjiang, China; ^3^University of Chinese Academy of Sciences, Beijing, China; ^4^Xinjiang Institute of Ecology and Geography, Chinese Academy of Sciences, Urumqi, China

**Keywords:** desert ecosystem, refixation, carbon balance, water use strategy, stem recycling photosynthesis

## Abstract

Water availability could alter multiple ecophysiological processes such as water use strategy, photosynthesis, and respiration, thereby modifying plant water use and carbon gain. However, a lack of field observations hinders our understanding of how water availability affects stem photosynthesis at both organ and plant levels of desert shrubs. In this study, we measured gas exchange and oxygen stable isotopes to quantify water sources, stem recycling photosynthesis, and whole-plant carbon balance in two coexisting *Haloxylon* species (*Haloxylon ammodendron* and *Haloxylon persicum*) at different groundwater depths in the Gurbantonggut Desert. The overall aim of the study was to analyze and quantify the important role of stem recycling photosynthesis for desert shrubs (*Haloxylon* species) under different groundwater depths. The results showed that (1) regardless of changes in groundwater depth, *H. ammodendron* consistently used groundwater and *H. persicum* used deep soil water as their main water source, with greater than 75% of xylem water being derived from groundwater and deep soil water for the two species, respectively; (2) stem recycling photosynthesis refixed 72–81% of the stem dark respiration, and its contribution to whole-plant carbon assimilation was 10–21% for the two species; and (3) deepened groundwater increased stem water use efficiency and its contribution to whole-plant carbon assimilation in *H. persicum* but not in *H. ammodendron*. Our study provided observational evidence that deepened groundwater depth induced *H. persicum* to increase stem recycling photosynthetic capacity and a greater contribution to whole-plant carbon assimilation, but this did not occur on *H. ammodendron.* Our study indicates that stem recycling photosynthesis may play an important role in the survival of desert shrubs in drought conditions.

## Introduction

Leaves are widely accepted to be the primary organ where photosynthetic carbon assimilation occurs in plants (White et al., 2016). However, there is mounting evidence challenging this paradigm because stems can also assimilate carbon, providing an alternative and significant carbon source in plants (Saveyn et al., 2010; Bloemen et al., 2013a; Cernusak and Cheesman, 2015; Henry et al., 2020). Stem photosynthesis has been reported in a wide variety of tree species, with instantaneous rates ranging from 0.5 to 9 μmol m^–2^ s^–1^, and although mostly this is refixation of respiratory CO_2_, stems rarely achieve net positive assimilation (Nilsen, 1995; Pfanz et al., 2002; Ávila et al., 2014; Vandegehuchte et al., 2015). The CO_2_ fixed by stem photosynthesis is derived from either stems themselves through stem respiration in living cells or belowground root respiration, which subsequently transports upward *via* the transpiration stream (Bloemen et al., 2013b). Accumulating evidence has suggested that stem photosynthesis is an additional mechanism of carbon sequestration across biomes (Ávila et al., 2014; Vandegehuchte et al., 2015; De Roo et al., 2020).

With episodes of drought events increasing across most of the world (IPCC, 2019), photosynthetic stems may become an advantage for maintaining positive carbon balance at the whole-plant level, such as high water and carbon use efficiency. For example, up to 97% of the CO_2_ released from young branches of birch (*Betula pendula* Roth.) was refixed *via* stem photosynthesis (Wittmann et al., 2006). Similarly, young *Eucalyptus globules* seedlings have an increased capacity to refix internally respired CO_2_ (up to 96%) (Eyles et al., 2009). Cernusak and Marshall (2000) demonstrated that the water use efficiency of gross photosynthesis in Western white pine was 50 times higher in the bark than in the leaves. Additionally, stem photosynthesis may also help in the maintenance of hydraulic functioning, too (Sevanto, 2014; Ávila and Tezara, 2018). A recent study showed that stem photosynthesis can also drive bark water uptake to refill embolized vessels in excised *Salix matsudana* branches that are submerged in deionized water (Liu et al., 2019). Thus, it can be seen that stem photosynthesis plays an important role during drought stress and may become important for plant survival when leaf photosynthesis and phloem transport are limited (Cernusak and Cheesman, 2015; Wittmann and Pfanz, 2018; Hubeau et al., 2019; De Roo et al., 2020; Henry et al., 2020).

The Gurbantonggut Desert is the second largest desert in northwest China. The woody layer is dominated by *Haloxylon ammodendron* and *Haloxylon persicum* Bunge ex Boiss, which are phreatophytes and respond non-linearly to growing season precipitation (Dai et al., 2015). Both species of *Haloxylon* have a lower photosynthetic active area, as twigs replace leaves to perform photosynthetic functions (Xu et al., 2007; Zou et al., 2010). Most stems are lignified from twigs and have switched to twig-stem CO_2_ assimilation in drought stress after twigs senesce or abscission (Smith et al., 1996). As a result, stem photosynthesis may be an important, though often overlooked, component of plant carbon balance (Steppe et al., 2015; Vandegehuchte et al., 2015; Bloemen et al., 2016; Tarvainen et al., 2018; De Roo et al., 2020). Despite recent interest in stem photosynthesis and its effect on hydraulic integrity (Schmitz et al., 2012; Cernusak and Cheesman, 2015; Tarvainen et al., 2018; Liu et al., 2019; De Roo et al., 2020), less is known about how photosynthetic stems respond to drought in desert shrubby species.

This study aims to fill this knowledge gap by characterizing the stem carbon dynamics in *Haloxylon* species growing under two different groundwater depths. We measured two *Haloxylon* species native to the Gurbantonggut Desert *in situ*. Across two groundwater depths, stem gas exchange, environmental factors, and plant water sources were monitored with the goal of (1) assessing plant water use strategy; (2) comparing the differences in carbon exchange dynamics and water use efficiency of stems; (3) quantifying refixation of carbon released from dark respiration and the contribution of stem recycling photosynthesis to whole-plant carbon assimilation. We hypothesized that deeper groundwater depth will increase the contribution of stem assimilation at the whole plant level, as well as increase the ratio of stem area to leaf area or decrease leaf photosynthetic capacity. We hope to achieve a deep understanding of the impact of groundwater depth changes on stem photosynthesis and whole-tree carbon balance in desert ecosystems, resulting in better predictions under projected climatic change scenarios.

## Materials and Methods

### Study Site

This study was carried out near the Fukang Station of Desert Ecology and the southern periphery of the Gurbantonggut Desert in Central Asia. This region has a continental arid temperate climate, with a hot, dry summer and a cold winter. The air temperature ranges from a minimum of −42.2°C in winter to a maximum of 44.2°C in summer, and the annual mean temperature is 6.6°C. Annual mean precipitation is 163 mm, around 75% of which falls during the growing season (May–October). Precipitation prior to the growing season (November–April) is mostly in the form of snow, which covers the soil for most of this period and melts in April (Zhou et al., 2012). The soil type is desert solonetz at 0–100 cm depth, with eolian sandy soil on top. The groundwater depth ranges from 3 m in the Fukang Station of Desert Ecology to 16 m in the southern periphery of the Gurbantonggut Desert.

**FIGURE 1:**
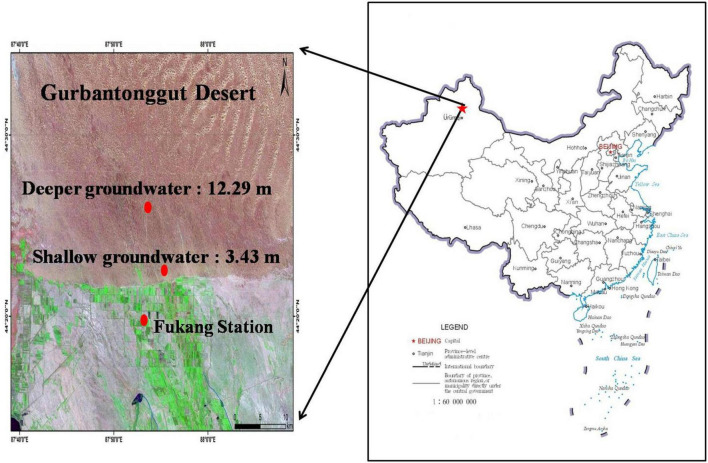
Location of the two experimental study sites. The shallow groundwater site has a groundwater depth of 3.43 m, and the deeper groundwater site has a groundwater depth of 12.29 m.

Two similar shrub-dominated desert communities were selected. Both sites are dominated by deep-rooted phreatophyte shrubs that depend primarily on groundwater for survival (Xu et al., 2007; Dai et al., 2015). The two sites were 13.5 km apart and located at the southern periphery of the Gurbantonggut desert, <7.5 km and <21.0 km away from Fukang Desert Station, and are dominated by *H. ammodendron* and *H. persicum*. The groundwater depth is 3.43 ± 0.02 m and 12.29 ± 0.19 m at these two sites. Throughout this study, we refer to the site with a groundwater depth of 3.43 m as a shallow groundwater site, and the site with a groundwater depth of 12.29 m as a deeper groundwater site (Figure 1).

### Leaf and Stem Recycling Photosynthetic Measurements

All photosynthetic measurements were carried out on clear days during July–August 2020. The photosynthetic light-response curve (09:00–14:00) and diurnal variation in instantaneous gas exchange rates (08:00–18:00) of stems were measured using one portable open gas exchange system (Li-6400XT, Li-Cor Inc., Lincoln, NE, United States) on consecutive campaigns of 2–3 days. For stem photosynthetic light-response curves, photosynthetic photon flux density (PPFD) was supplied by a 15 × 7 cm stem chamber with a red-blue light source (*P*-Chamber, MilletHill Biotech Co. Ltd., Shanghai, China) (Chang et al., 2020) and was reduced in a stepwise manner at 10 PPFD levels (2,000, 1,600, 1,200, 800, 400, 200, 150, 100, 50, and 0 μmol m^–2^ s^–1^), and the other conditions in the chamber were set to 400 ppm and 1.5 kPa for CO_2_ and atmospheric vapor pressure deficit (VPD), respectively. At each site, three stems with a diameter of 8–10 mm were selected for measurement in light and dark conditions. Each stem was allowed 6–8 min to equilibrate at each PPFD, and before measurements were recorded. Stem recycling photosynthetic light-response curve parameters were estimated using a modified rectangular hyperbola model (Equation 1):


(1) $$P_{g}=\alpha \frac{\left(1-\beta I\right)}{\left(1+\gamma I\right)}-R_{d}$$

where *P*_*g*_ is the gross photosynthetic rate of the stem (including dark respiration), *I* is the PPFD, *R*_*d*_ is the daytime dark respiration rate, α is the initial slope of the photosynthetic light-response curve where PPFD ≈ 0 μmol m^–2^ s^–1^, and β and γ are coefficients that are independent of *I* and were obtained by curve fitting (Ye and Yu, 2008).

The diurnal photosynthetic patterns of leaves and stems were measured simultaneously for each species and at each groundwater depth. Each measurement period of gas exchange lasted for approximately 10 h (between 08:00 and 18:00 h), and measurements were taken approximately every 2 h. A smart quantum sensor (MQ-500, Apogee, United States) was used to measure PPFDi at each measurement time point, and this value was manually entered into the Li-6400XT and *P*-Chamber to generate the corresponding irradiance. For the stem recycling photosynthetic rate (*P*_*stem*_), positive net assimilation is, however, rarely reached, in most studies. Stem recycling photosynthesis was therefore assumed to be equal to the difference between the gas exchange rate under light (*R*_*l*_) and dark (*R*_*d*_) (Equation 2; Wittmann et al., 2006):


(2) $$P_{stem}=\left|R_{d}-R_{l}\right|$$

Additionally, as stem recycling photosynthesis refixes respiratory losses from the stem, refixation was estimated using *R*_*d*_ and *R*_*l*_ as shown in the following equation (Cernusak and Marshall, 2000):


(3) $$\%R\text{efixation}=\frac{\left|R_{d}-R_{l}\right|}{R_{d}}$$

To ensure the accuracy of stem recycling photosynthetic rate measurements, we used another method for cross-validation. A custom-made transparent polycarbonate chamber (15 cm × 7 cm × 7 cm) was attached to the stem, and CO_2_ and H_2_O fluxes inside the chamber were measured using a portable, open gas exchange system (Li-840A, Li-Cor Inc., Lincoln, NE, United States). Measurements using the transparent chamber directly obtained the net gas exchange rate. To estimate the dark respiration rate, we covered the chamber with an opaque canvas cover, allowing stem recycling photosynthetic rate to be calculated as the difference between measurements in the light and the dark (Supplementary Figure 1). The detailed procedures are described by Huang and Li (2015).

### Determining Water Use Strategies of Two Species

Oxygen stable isotope (δ^18^O) measurements were used to determine the water use strategy of both *Haloxylon* species at different groundwater depths. For each species at each site, four healthy plants with average canopy size were randomly selected. Plant xylem and soil samples were collected simultaneously on three consecutive clear days. Only sunlit twigs from each plant were sampled. Excised leaves were quickly decorticated and placed in screw-cap glass vials that were sealed using Parafilm and stored in a freezer until subsequent extraction of the xylem water for δ^18^O analysis. Three soil cores were collected next to the sampled plants. Soil samples were collected at 20 cm intervals from the topsoil to the groundwater table. Four replicate soil samples at each layer were sampled and sealed in glass vials and frozen for soil water extraction and δ^18^O analysis.

Xylem water and soil water were extracted using cryogenic vacuum distillation, and the extracted water was stored in sealed glass vials at 2^°^C. The oxygen isotopic composition of the water was determined using a liquid water isotope analyzer (LWIA, DLT-100, Los Gatos Research Inc., Mountain View, CA, United States). Two methods were used to determine the water use of the two species. One was the direct inference approach, which compares δ^18^O composition between groundwater, the soil water profile, and xylem water by plotting them together. The other method was the IsoSource model, which determines the possible range of different water sources used by plants (Phillips and Gregg, 2003). Based on the similarities in the δ^18^O values of soil water, the soil profile was divided into two sections, and the following two potential water sources were finally identified using a post-integrated method: (1) deep soil layer, which was the intermediated section between the upper soil layer (0–1 m) and the near groundwater layer; and (2) groundwater layer (0–1.5 m above the groundwater depth), which could be identified by a significant increase in soil water content (SWC) and has a similar isotopic composition to that of groundwater [For details on the application of the IsoSource model, refer to Liu et al. (2016) and Wu et al. (2019)].

### Stem Water Use Efficiency and Environmental Measurements

Stem water use efficiency was calculated using the ratio of stem recycling photosynthesis rate to transpiration rate (μmol CO_2_ m^–2^ s^–1^/mmol H_2_O m^–2^ s^–1^). Air temperature (*T*_*air*_) (*^o^*C) and relative humidity (RH) (%) were measured at two sites every 10 s and averaged at 30 min intervals using a portable weather station (S-THB-M008, Onset, Bourne, MA, United States). Values of *T*_*air*_ and RH were used to calculate the atmospheric VPD. A TDR 150 probe was used to measure soil temperature and volumetric water content at 5 and 10 cm soil depths (FieldScout TDR 150, spectrum Technologies, Aurora, IL, United States). To quantify the contribution of stem recycling photosynthesis to the entire plant, plants were destructively harvested at the end of all measurements and measured for total stem surface area and total leaf area using Vernier calipers and a leaf area meter (Li-3100, Li-Cor Inc., Lincoln, NE, United States), respectively. In this study, we used the following equation to calculate the contribution of stem photosynthesis to the entire plant:


(4) $$\%P_{stem}=\frac{P_{stem}\times S_{stem}}{P_{stem}\times S_{stem}+P_{leaf}\times S_{leaf}}$$

where *P*_*stem*_ is stem recycling photosynthesis rate, *S*_*stem*_ is total stem surface area, *P*_*leaf*_ is net leaf photosynthesis rate, and *S*_*leaf*_ is total leaf surface area.

### Statistical Analyses

All descriptive statistics and statistical analyses (including linear regression, one-way ANOVA, paired *t*-test, independent samples *t*-test) were conducted using the R software (version 3.3.1) (R Core Team, 2020). Given the small sample size (*n* = 3–36), significance levels of 0.05 and 0.10 were used to represent strong and moderate effects, respectively. All data visualization was performed using the Origin 2017 software package (OriginLab Corporation Northampton, MA, United States).

## Results

### Meteorological and Groundwater Conditions in the Two Communities

**FIGURE 2:**
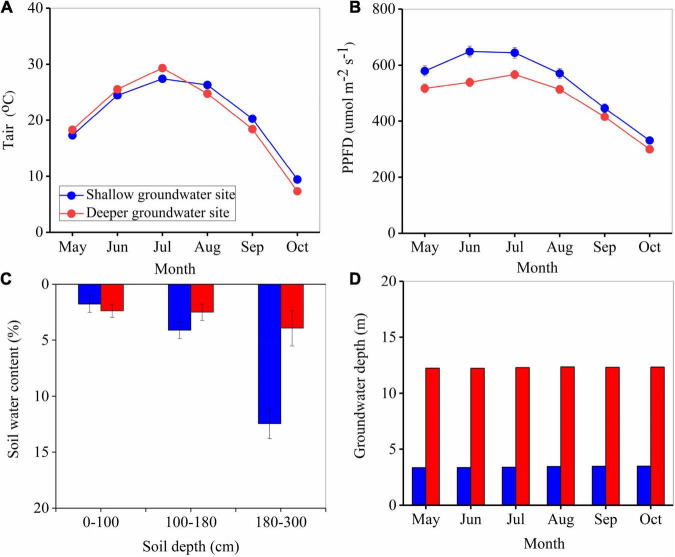
Variation in air temperature (*T*_*air*_) **(A)**, photosynthetic photon flux density (PPFD) **(B)**, soil water content **(C)**, and groundwater depth **(D)** during the growing season at the two study sites during 2020.

The variation in the major meteorological conditions and groundwater depths in two sites during the growing season of 2020 are presented in Figure 2. Monthly average daily *T*_*air*_ and PPFD followed similar patterns at the two sites (*P* > 0.05) (Figures 2A,B), with maximum daily temperatures ranging from 27.5 to 29.0° Cand PPFD ranging from 567 to 645 μmol m^–2^ s^–1^. Deeper groundwater depth resulted in lower soil water content, especially in 1.8–3.0 m soil layer, with values of 12.47 and 3.94% at two sites (Figure 2C). There was no significant difference in VPD between the two sites, with an average value of 1.67 kPa. In contrast, groundwater depths varied significantly between the two sites (*P* < 0.01) (Figure 2D), with an average groundwater depth of 3.43 ± 0.02 and 12.29 ± 0.02 for the shallow and deeper groundwater sites, respectively.

### Water Use Strategy of Two *Haloxylon* Species

**FIGURE 3:**
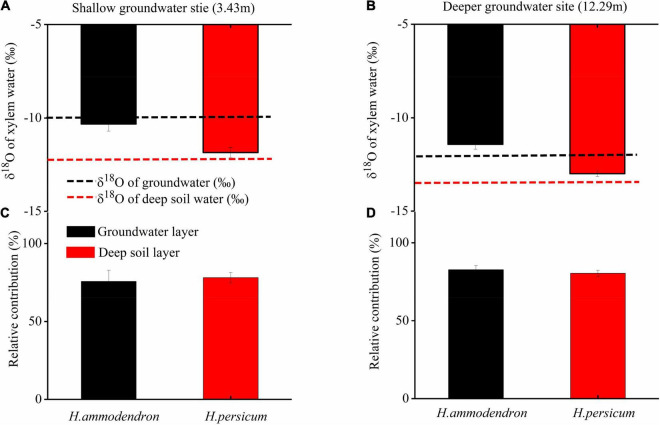
Comparison of the oxygen stable isotope ratio (δ^18^O) of xylem water and deep soil water and groundwater **(A,B)**, and the relative contribution of different water sources **(C,D)** for the two *Haloxylon* species at two sites.

Variation in the water use strategies of the two *Haloxylon* species in two sites was used to determine if they may explain variability in stem photosynthesis. The values of δ^18^O of xylem, each soil layer, and groundwater are shown in Figure 3 and Supplementary Figure 2. Regardless of changes in groundwater depth, *H. ammodendron* used similar water sources; the value of δ^18^O of xylem water was much closer to the value of δ^18^O of groundwater (Figures 3A,B): greater than 75% of the xylem water was derived from groundwater (Figures 3C,D). Similarly, *H. persicum* used deep soil water as its main water source; greater than 78% of the xylem water was derived from deep soil layers (Figure 3).

### Photosynthetic Response to Light of Stems

**FIGURE 4:**
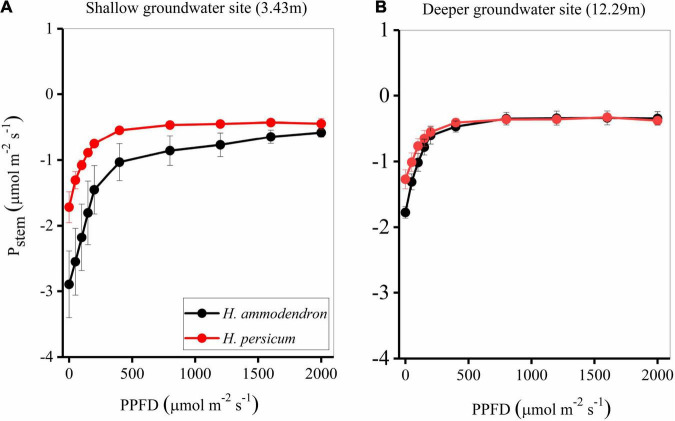
Photosynthetic light responses as measured *in situ* for stems **(A,B)** of the two *Haloxylon* species at two sites. Values are means of three replicates ± SE of the mean.

Stems of both *Haloxylon* species became light-saturated at approximately 1,000 μmol m^–2^ s^–1^ PPFD (Figures 4A,B and Table 1). Both species had a lower *R*_*d*_ and maximum *P*_*max*_ at deeper groundwater sites, with the values of −1.80 and −0.31 μmol m^–2^ s^–1^ for *H. ammodendron* and −1.29 and −0.32 μmol m^–2^ s^–1^ for *H. persicum*. In contrast, the two species had a higher initial slope (α) at a deeper groundwater site (Figures 4A,B and Table 1).

**TABLE 1:** Stem light response parameters for two *Haloxylon* species growing under different groundwater depths.

	Site	LSP	R_*d*_	P_*max*_	α
*H. ammodendron*	1	1272 (10)	2.98 (0.53)	−0.55 (0.04)	0.0136 (0.0015)
	2	1131 (89)	1.80 (0.09)	−0.31 (0.09)	0.0155 (0.0025)
*H. persicum*	1	989 (46)	1.81 (0.78)	−0.39 (0.16)	0.0088 (0.0025)
	2	1089 (196)	1.29 (0.15)	−0.32 (0.04)	0.0092 (0.0004)

*Site 1, shallow groundwater depth; Site 2, deeper groundwater depth; LSP, light saturation point (μmol m^–2^ s^–1^); R_d_, dark respiration (μmol m^–2^ s^–1^); P_max_, light-saturated CO_2_ uptake (μmol m^–2^ s^–1^) was derived by applying a modified rectangular hyperbola model to light response data; α, initial slope. The values in parentheses represent SE (n = 3).*

### Diurnal Variations in Stem Recycling Photosynthesis and Water Use Efficiency

**FIGURE 5:**
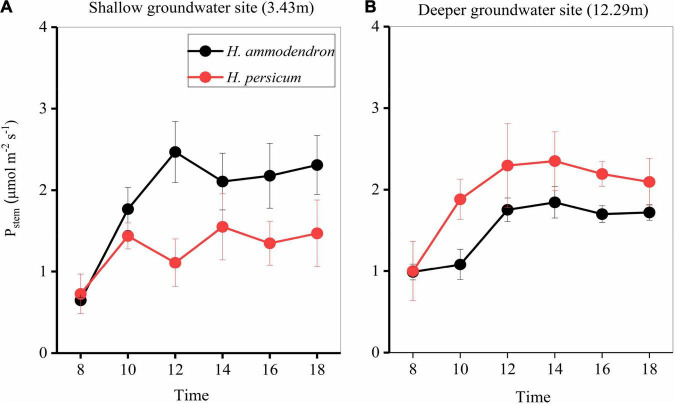
Average daily patterns of the stem (*P*_*stem*_) **(A,B)** photosynthesis for two *Haloxylon* species at two sites. Error bars represent SE (*n* = 3).

Throughout a diurnal time course, at a deeper groundwater site, the stem recycling photosynthetic rate of *H. persicum* was increased, with the maximum *P*_*stem*_ recorded being 1.84 vs. 2.35 μmol m^–2^ s^–1^ at shallow and deeper groundwater sites, respectively (Figures 5A,B). However, the opposite pattern was observed in *H. ammodendron*, with the maximum *P*_*stem*_ recorded being 2.47 vs. 1.55 μmol m^–2^ s^–1^ at each of the sites (Figures 5A,B).

**FIGURE 6:**
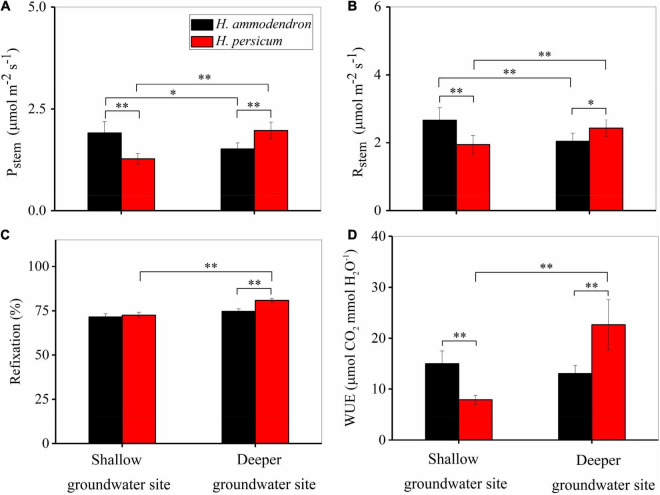
Comparison of daily average stem (*P*_*stem*_) photosynthesis **(A)**, stem dark respiration (*R*_*stem*_) **(B)**, stem refixation **(C)** (expressed as a percentage of *P*_*stem*_ and *R*_*stem*_), and stem water use efficiency **(D)** for two *Haloxylon* species at two sites. Error bars represent SE (*n* = 18). *P < 0.1, **P < 0.05.

On average, *H. ammodendron* had significantly lower values of *P*_*stem*_ (1.91 vs. 1.51 μmol m^–2^ s^–1^) and *R*_*stem*_ (2.67 vs. 2.04 μmol m^–2^ s^–1^) at the two sites (Figures 6A,B). The values of *P*_*stem*_ and *R*_*stem*_ of *H. persicum* increased up to 57% at the deeper groundwater site (*P* < 0.05) (Figures 6A,B). Furthermore, the amount of respired CO_2_ refixed by stem photosynthesis ranged from 72 to 81% in the two *Haloxylon* species at two sites (Figure 6C). Deeper groundwater depth resulted in higher stem water use efficiency of *H. persicum*, with the value of 7.90 vs. 22.61 μmol CO_2_ mmol H_2_O^–1^, but *H. ammodendron* remained unchanged 14.90 vs. 13.01 μmol CO_2_ mmol H_2_O^–1^ (Figure 6D).

### The Contribution of Stem Recycling Photosynthesis to Whole-Plant Carbon Assimilation

**FIGURE 7:**
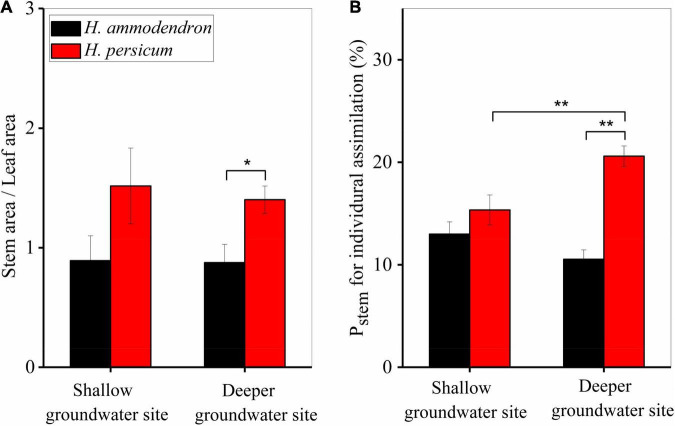
Variations in stem area per leaf area **(A)** and the contribution of stem photosynthesis to whole-plant carbon assimilation **(B)** for two *Haloxylon* species growing at two sites. Error bars represent SE (*n* = 3). *P < 0.1, **P < 0.05.

The ratio of stem area to leaf area (stem area/leaf area) of the two *Haloxylon* species at two sites was determined using destructive sampling (Figure 7A). The proportion of stem area in *H. persicum* was significantly higher than in *H. ammodendron*, but there was no difference within species between sites (Figure 7A). We estimated the contribution of stem recycling photosynthesis to whole-plant carbon assimilation to be at least 10% for *H. ammodendron* and to be as high as 21% for *H. persicum* at a deeper groundwater site (Figure 7B).

## Discussion

**FIGURE 8:**
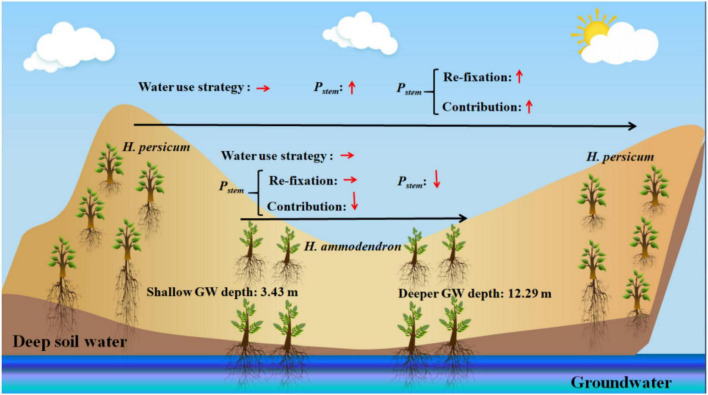
Conceptual diagram illustrating the main results of this study. GW, groundwater.

Understanding the role of stem recycling photosynthesis in two *Haloxylon* species growing in habitats differing in groundwater depth is an important step toward improving predictions of carbon cycle dynamics under drought stress in arid and semiarid ecosystems (Ávila et al., 2014; Cernusak and Cheesman, 2015; Tarvainen et al., 2018; De Roo et al., 2020). In this study, we monitored the responses of stem recycling photosynthesis under an exceptionally wide range of groundwater depths from 3.43 to 12.29 m. We found that the deep soil water use strategy can induce the increase in stem recycling photosynthesis capacity of *H. persicum* when exposed to a deeper groundwater depth. We also found that stem recycling photosynthesis of *H. ammodendron* was shaped by *T*_*air*_ and SWC, and stem recycling photosynthesis of *H. persicum* showed a positive correlation with *P*_*leaf*_ (Figure 8).

Plant water use strategy is particularly important when there is a change in plant community structure that might influence the accessibility of shallower vs. deeper soil water pools or groundwater (Campos et al., 2013; Scott et al., 2014). In this study, the isotopic analysis showed that variation in groundwater depth did not affect the water use strategies of either *Haloxylon* species (Figure 3). *H. ammodendron* consistently used a greater fraction of groundwater as its main water source, while the largest contributor for *H. persicum* was the deeper soil water pool. Previous root-system investigation experiments characterized both of these species as being deep-rooted plants (Zou et al., 2010). The different water use strategies of *Haloxylon* species may be related to the microtopography where they are distributed. *H. ammodendron* mainly grows at the bottom of sand dunes, and its roots could penetrate up to 10 m below the surface (Xu et al., 2007). This deep rooting depth enables it to access groundwater (Lv et al., 2013; Dai et al., 2015; Wu et al., 2019). Unlike *H. ammodendron*, *H. persicum* usually grows on dune crests, placing it farther from the groundwater table. Excessive energy costs might prevent its roots from accessing groundwater (Tiemuerbieke et al., 2018). Therefore, *H. persicum* depends on deep soil water, which is derived from infiltration of precipitation and groundwater fluctuations (Figure 3).

Even at places where the groundwater was deeper, neither *Haloxylon* species changed its water use strategy, though photosynthesis of stems did respond to groundwater depth (Figures 4, 5). Stems had significantly lower light-saturated PPFD and maximum stem photosynthetic rates. The observed value of light-saturated stem photosynthesis corresponds to measurements reported for stems of most other woody species, which range from 200 to 400 μmol m^–2^ s^–1^ (Wittmann and Pfanz, 2008; Eyles et al., 2009). The maximum stem recycling photosynthetic rate was 1.58 ± 0.3 μmol m^–2^ s^–1^, which is consistent with reports for other species [0.1–9 μmol m^–2^ s^–1^, reviewed by Ávila et al. (2014)]. Surprisingly, deeper groundwater sites had a strong positive effect on stem recycling photosynthesis of *H. persicum*, but this did not occur on *H. ammodendron* (Figures 5–7). This could be attributed to the fact that the response of *H. ammodendron* to water changes mainly relies on morphological regulation to maintain the stability of physiological activities (Xu et al., 2007; Zou et al., 2010). The results partially support our initial hypothesis that stem photosynthesis would contribute more to the organ and whole-plant carbon balance of two species under deeper groundwater depths.

Stem recycling photosynthesis does not depend on the uptake of atmospheric CO_2_ but can instead rely on internal dark respiration (Cernusak and Marshall, 2000). Our results also suggested that stem recycling photosynthesis refixes CO_2_ released during stem dark respiration, resulting in 72–81% of respired CO_2_ being refixed (Figure 6). The observed refixation value corresponds to observations reported for stems recycling photosynthesis of most other woody species, which suggest that this recycling mechanism can offset between 7 and 98% of stem dark respiration (Wittmann et al., 2006; Ávila et al., 2014; De Roo et al., 2020). Refixation has two advantages over leaf photosynthesis: there is very less associated water loss and refixing respired carbon increases carbon use efficiency, which increases stand-level productivity (Vandegehuchte et al., 2015). Stems always have higher water use efficiency (Cernusak and Marshall, 2000); we found that two *Haloxylon* species’ water use efficiency of stems was 3–5 times higher than that of the leaves (Supplementary Table 1). This may be due to the fact that bark lenticels are far less than leaf stomata. Additionally, stem recycling photosynthesis can be accomplished with stomata closed as it can refix internally respired CO_2_ (Wittmann et al., 2006; Cernusak and Hutley, 2011).

Although stem recycling photosynthesis has been widely studied for its effects on tree carbon cycling (Nilsen, 1995; Pfanz et al., 2002; Ávila et al., 2014; De Roo et al., 2020), it has surprisingly been neglected on its contribution to the whole-plant carbon assimilation. We estimated that stem recycling photosynthesis contributed 10–21% of the total whole-plant carbon assimilation for the two *Haloxylon* species (Figure 7). Previous studies have shown that stem photosynthesis accounted for 10–15% of whole-tree carbon input in *Populus tremula* during the summer (Kharouk et al., 1995; Pfanz et al., 2002). In mature *Eucalyptus miniata* trees, 10% of branch diameter growth was fueled by stem photosynthesis (Cernusak and Hutley, 2011). Altogether, the results indicated that two *Haloxylon* species, especially *H. persicum*, exhibited higher stem recycling photosynthesis, which plays an important role in desert plant carbon balance (Cernusak and Marshall, 2000; Eyles et al., 2009; Liu et al., 2019).

## Conclusion

Our study quantified the relative importance of changes in stem recycling photosynthetic capacity across different groundwater depths. Different water use strategies employed by the two *Haloxylon* species drove *H. persicum*, but not *H. ammodendron*, to increase its photosynthetic capacity when groundwater was deeper. Furthermore, deeper groundwater also resulted in a higher contribution of stem recycling photosynthesis to whole-plant carbon assimilation and stem water use efficiency, indicating that water use and carbon gain become more efficient under drought conditions. Ecophysiological studies on stem photosynthesis of woody plants are still very much under-explored. Future studies on different desert woody plants should also include characterizing how stem photosynthesis may impact the resistance and resilience of those species to drought. Accurate quantification of the capacity for refixation of respired carbon by stem photosynthesis and its contribution to the whole plant may reveal that the contribution of stem photosynthesis to the total carbon budget of desert ecosystems is significant.

## Data Availability Statement

The raw data supporting the conclusions of this article will be made available by the authors, without undue reservation.

## Author Contributions

RL, YW, CL, and YL planned and designed the research. RL, XF, and JM collected the data. RL and XF analyzed the data. RL, XF, and CL interpreted the results and wrote the manuscript. All authors contributed critically to the drafts.

## Conflict of Interest

The authors declare that the research was conducted in the absence of any commercial or financial relationships that could be construed as a potential conflict of interest.

## Publisher’s Note

All claims expressed in this article are solely those of the authors and do not necessarily represent those of their affiliated organizations, or those of the publisher, the editors and the reviewers. Any product that may be evaluated in this article, or claim that may be made by its manufacturer, is not guaranteed or endorsed by the publisher.
