# Supplementary material for: The Importance of Stem Photosynthesis for Two Desert Shrubs Across Different Groundwater Depths
Source: Front Plant Sci. 2022 Mar 10;13:804786. doi: 10.3389/fpls.2022.804786 (PMC8965657; doi:10.3389/fpls.2022.804786)
Supplement: Supplementary file 1 [file Data_Sheet_1.pdf]

Supplemental files

Table S1 Comparison of leaf and stem water use efficiency under different groundwater depth

	Site	WUE_{leaf}	WUE_{stem}
H. ammodendron	1	7.94 (1.26)	14.98 (2.53)
	2	3.96 (0.34)	13.07 (1.63)
H. persicum	1	5.11 (0.78)	7.91 (0.88)
	2	4.48 (0.45)	22.61 (4.93)

Site 1: shallow groundwater depth, *Site 2*: deeper groundwater depth. The value in parentheses was represented SE (n=18).

Fig. S1

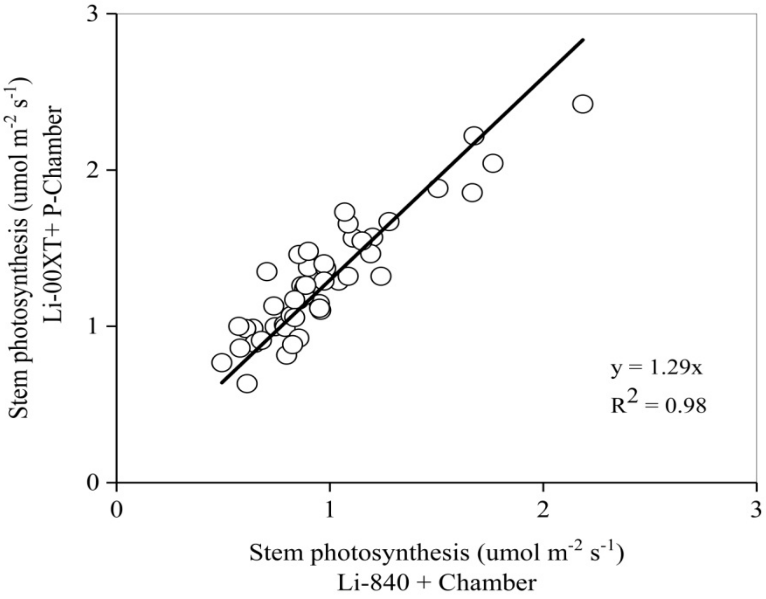

Fig. S1 Relationship of stem photosynthesis measured between Li-840 + Chamber and Li-6400XT + *P*-Chamber in 2020-2021 growing season

Fig. S2

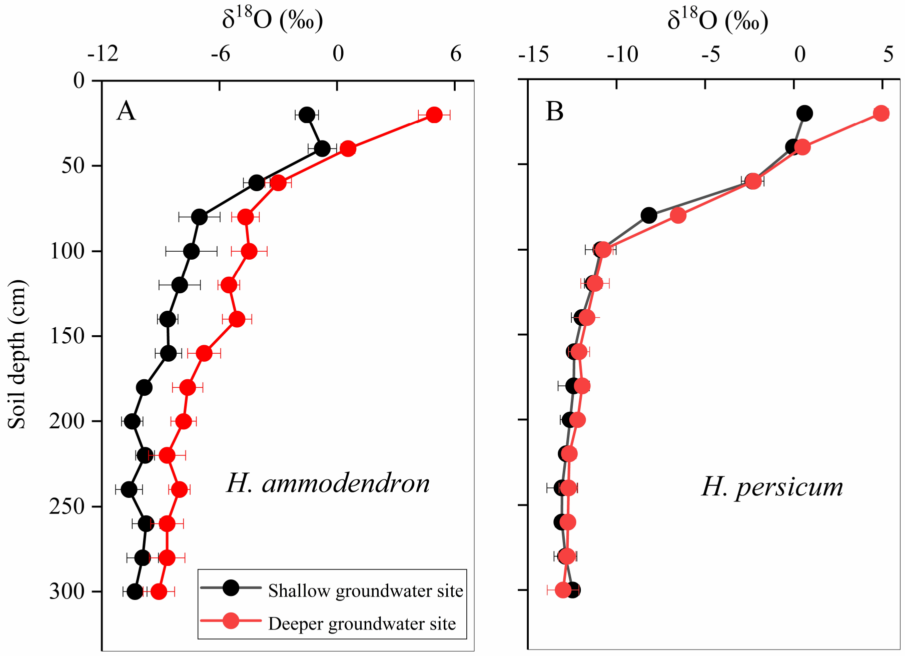

Fig. S2 The $\delta^{18}O$ values of soil water for H. ammodendron and H. persicum at two groundwater sites. Error bars represent the standard errors of the mean (n=3).
